# Comparative Analysis of Citrus Species’ Flavonoid Metabolism, Gene Expression Profiling, and Their Antioxidant Capacity under Drought Stress

**DOI:** 10.3390/antiox13091149

**Published:** 2024-09-23

**Authors:** Muhammad Junaid Rao, Mingzheng Duan, Momina Eman, Huwei Yuan, Anket Sharma, Bingsong Zheng

**Affiliations:** 1 State Key Laboratory of Subtropical Silviculture, College of Forestry and Biotechnology, Zhejiang A & F University, Hangzhou 311300, China; mjr@zafu.edu.cn (M.J.R.); hwyuan@zafu.edu.cn (H.Y.); anketsharma@gmail.com (A.S.); 2 College of Agronomy and Life Sciences, Zhaotong University, Zhaotong 657000, China; 3 Key Laboratory of Horticultural Plant Biology, College of Horticulture and Forestry, Huazhong Agricultural University, Wuhan 430070, China; emomina007@gmail.com; 4 Institute of Pure & Applied Biology (IP&AB), Bahauddin Zakariya University, Multan 60800, Punjab, Pakistan

**Keywords:** flavonoids, gene expression, drought stress, citrus, antioxidant capacity

## Abstract

Citrus species are widely cultivated across the globe and frequently encounter drought stress during their growth and development phases. Previous research has indicated that citrus species synthesize flavonoids as a response mechanism to drought stress. This study aimed to comprehensively quantify and analyze the presence of 85 distinct flavonoids in the leaf and root tissues of lemon (drought susceptible) and sour orange (drought tolerant). In drought-stressed sour orange roots, flavonoids, such as isosakuranin, mangiferin, trilobatin, liquiritigenin, avicularin, silibinin, and glabridin, were more elevated than control sour orange roots and drought-stressed lemon roots. Additionally, hydroxysafflor yellow A, cynaroside, tiliroside, and apigenin 7-glucoside were increased in drought-stressed sour orange leaves compared to drought-stressed lemon leaves. Under drought stress, flavonoids such as (-)-epigallocatechin, silibinin, benzylideneacetophenone, trilobatin, isorhamnetin, 3,7,4′-trihydroxyflavone, and liquiritigenin were significantly increased, by 3.01-, 3.01-, 2.59-, 2.43-, 2.07-, 2.05-, and 2.01-fold, in sour orange roots compared to control sour orange roots. Moreover, the total flavonoid content and antioxidant capacity were significantly increased in drought-stressed sour orange leaves and root tissues compared to drought-stressed lemon leaves and root tissues. The expression levels of genes involved in flavonoid biosynthesis were highly expressed in sour orange leaves and roots, compared to lemon leaves and root tissues, post-drought stress. These findings indicate that lemons fail to synthesize protective flavonoids under drought conditions, whereas sour orange leaves and root tissues enhance flavonoid synthesis, with higher antioxidant activities to mitigate the adverse effects of reactive oxygen species generated during drought stress.

## 1. Introduction

Environmental stresses, such as drought, salinity, high temperatures, and frost, annually cause significant economic losses in the agricultural sector [1,2]. Among these, drought is a critical factor, adversely affecting global agricultural production. Drought stress profoundly influences plant growth and agricultural yields by modifying plant metabolic processes [3]. It disrupts photosynthesis, cellular homeostasis, water and solute absorption, and the electron transport chain [4]. This disruption results in the overproduction of reactive oxygen species (ROS), leading to oxidative stress in citrus leaves and roots. The accumulation of ROS not only damages the photosynthetic machinery, but also interacts with DNA, proteins, and cell membranes, inhibits enzyme functions, and may lead to cell death [5]. In response to water deficit, plants employ dehydration avoidance or tolerance mechanisms, which include physiological adaptations, such as reducing water loss through cell wall hardening, accumulating osmoprotectant solutes and proteins, undergoing metabolic changes, and detoxifying ROS. Citrus plants, especially those cultivated in rain-fed conditions in semi-arid regions, are highly vulnerable to drought stress [6,7]. The severity of drought stress has also been linked to the lowest fruit quality observed in the past decade, characterized by increased fruit drop and defects, such as cracking and creasing, which substantially reduce the market value of fruits. Consequently, the overall fruit yield has declined by 70%. To mitigate these effects in semi-arid, citrus-growing areas, it is advisable to cultivate drought-resistant citrus varieties or utilize resilient rootstocks [8].

Citrus fruits are among the most significant agricultural crops, grown extensively across both subtropical and tropical areas, in over 140 countries globally. Major producers include China, Brazil, the USA, and India [9]. The range of citrus fruits cultivated commercially encompasses varieties such as pummelos, oranges, lemons, limes, and mandarins. Many of these, including wild mandarins and pummelos, originate from China [10]. Citrus crops face numerous environmental and biological challenges that can decrease production by 50% to 100% [2,11,12]. To cope with these challenges, citrus varieties generate numerous metabolites essential for their growth, defense, and development processes. Research indicates that cultivated citrus species increase the production of secondary metabolites in response to both biotic and abiotic stressors [13]. Citrus leaves show elevated flavonoid levels to mitigate the adverse effects of water scarcity [14]. Drought-resistant citrus rootstocks, like sour orange and *Poncirus trifoliata*, exhibit high metabolite concentrations, while drought-sensitive species, such as pummelos and lemons, have lower metabolite levels [12,15]. Flavonoid compounds are noted for their strong antioxidant properties and roles in signaling, reproduction, antimicrobial defense, and the regulation of cellular physiology in plants [16].

Flavonoids, a key group of secondary metabolites, have been recognized for their role in shielding cellular organelles by neutralizing ROS generated during both abiotic and biotic stresses in numerous plants [17]. Flavonoids, found in plant vacuoles, are known for their potent antioxidant capabilities, because they are rich in catechol structures that help to nullify ROS produced under stress [18]. Phenolic compounds, like anthocyanins and proanthocyanidins, also demonstrate substantial antioxidant properties and are crucial in shielding subcellular structures from both abiotic and biotic stresses [19,20]. Citrus leaves synthesize a variety of secondary metabolites that serve as a secondary line of defense by detoxifying the ROS arising from various stresses [14]. It has been recently observed that citrus species with high levels of flavonoids or those that quickly synthesize flavonoids following drought stress are better able to cope with such conditions compared to those with fewer metabolites or delayed flavonoid synthesis [16]. Despite this, the focus over the last decade has predominantly been on the rootstock/scion combination, biochemical parameters, antioxidant activities, and nutritional analysis of cultivated citrus varieties; the study of flavonoid quantification in leaves and roots in response to drought stress is relatively less explored in cultivated citrus species.

In our study, we examined two types of citrus species: sour orange, which is drought tolerant, and lemon, which is drought sensitive. We analyzed various flavonoid classes under drought conditions in both the leaf and root tissues of these species. Additionally, we assessed the antioxidant activities and gene expression related to flavonoid biosynthesis during drought stress. Physiological and biochemical measurements were performed at multiple stages of drought stress. This study enhances our understanding of specific flavonoid compounds in citrus roots and leaves, their regulation under drought conditions, and their implications for further research into drought resistance.

## 2. Materials and Methods

Sour orange (*Citrus aurantium*) and lemon (*Citrus limon*) seeds were grown under controlled conditions (10,000 LUX light intensity, 27 ± 1 °C temperature, and 65 ± 5% humidity) in a growth chamber. A normal amount of water and fertilizer were administered to each plant [14]. The seeds of the citrus species were collected from the Institute of Citrus Science, Huazhong Agricultural University. Six-month-old seedlings were selected for the drought stress treatment, including the control (without stress), 7 days of drought stress (7 DS), and 14 days of drought stress (14 DS). All the plants that were of the same age and size were selected for the drought treatment and water was withheld for 14 days. According to the root and leaf stress phenotype, we collected the control and 14 DS root and leaf samples (three replicates per species, each replicate had 7 leaves) for LC-MS/MS (liquid chromatography with tandem mass spectrometry) to determine the flavonoid metabolites. The control and drought-stressed roots were harvested from well-watered citrus plant and after 14 days of drought stress. For the gene expression analysis and the biochemical parameters, the leaf samples were collected from the control, 7 DS, and 14 DS plants. Each time, seven fully expanded leaves were harvested from the middle of the citrus plant and their fresh and dry weights were measured to calculate the relative water content.

### 2.1. RNA Isolation and Gene Expression Profiling

The RNA was extracted from 0.1 g of citrus root and leaf materials by using a NucleoSpin (Takara, Shanghai, China) RNA purification kit (https://www.takarabio.com/products/; accessed on 19 May 2024). A Vazyme R223-01 reverse transcriptase kit (Nanjing Vazyme Biotech., Nanjing, China) was used to synthesize the complementary DNA (cDNA) from 1 µg of citrus root and leaf total RNA materials. The expression analysis was performed using LightCycler 480 II, while the β-actin gene was used as an internal reference. The quantitative real-time polymerase chain reaction (q-PCR) primers of seven flavonoid biosynthesis genes, namely phenylalanine ammonia-lyase (PAL), cinnamate 4-hydroxylase (C4H), 4-coumarate-CoA ligase (4CL), TRANSPARENT TESTA 4 (TT4), TRANSPARENT TESTA 5 (TT5), TRANSPARENT TESTA 6 (TT6), and TRANSPARENT TESTA 7 (TT7), were categorized (Supplementary Table S3).

### 2.2. Citrus Root and Leaf Metabolic Profiling

The flavonoids within citrus root and leaf samples were analyzed using ultra-performance liquid chromatography–tandem mass spectrometry (UPLC-MS/MS). Liquid nitrogen was used to grind the freeze-dried citrus root and leaf materials for flavonoid analysis; 50 mg of sample powder was employed for both the qualitative and quantitative assessments. A total of 10 microliters of C_27_H_30_O_16_ (rutin) 4000 nmol/L was used as an internal standard. Then, 500 microliters of 70% aqueous CH₃OH methanol (Sigma-Aldrich, Shanghai, China) was added to each 50 mg of ground sample, followed by ultrasonic extraction (Model: KQ5200E) for 30 min at 5 °C. Centrifugation was performed at room temperature for 5 min at 12,200× *g*. The supernatant was collected and filtered using a 0.22 μm microporous filter (ANPEL, Shanghai, China) in a new centrifuge tube [21]. The filtered liquid was used for the UPLC-MS/MS analysis, while multiple reactions monitoring analysis, electrospray ionization–mass spectrometry (ESI-MS/MS), and UPLC analysis were performed as previously specified by Chen et al. [21,22]. The analytical conditions related to the UPLC instrument were as follows: UPLC column, Waters ACQUITY UPLC HSS T3 C18 (100 mm × 2.1 mm, 1.8 µm); solvent system, water with 0.05% HCOOH formic acid (A), C_2_H_3_N acetonitrile with 0.05% formic acid (B). The flow rate of the solvent system was 0.35 mL/min, the temperature was maintained at 40 °C, and the injection volume was 2 μL, as described by Chen et al. [21]. The ESI-MS/MS operating system was equipped with an ESI turbo ion spray interface, functioning in both positive and negative ion modes, and managed by the Analyst software, version 1.6.3 (https://sciex.com/products/software/analyst-software; accessed on 15 February 2024). The ESI source parameters were set as follows: ion source, ESI+/−; source temperature set to 550 °C; an ion spray voltage of 5500 V (positive ion mode) and −4500 V (negative ion mode); and curtain gas at 35 psi, as outlined by Chen et al. [21] (Supplementary File S1). Mass spectrum peaks were detected qualitatively and quantitatively, based on the retention times (RTs) and calibration curves of the standards (Supplementary Table S1).

### 2.3. DPPH Free Radical Scavenging Activity and Capacity

The assessment of the antioxidant activity and capacity of the citrus root and leaf samples was carried out using C_18_H_12_N_5_O_6_ DPPH (2,2-diphenyl-1-picrylhydrazyl) from Sigma-Aldrich, a free radical that can accept hydrogen from antioxidants [23]. A total of 0.1 g of root and leaf powder was ground in 1000 microliters of extraction solution, consisting of 70% C_2_H_6_O (ethanol), 29% water, and 1% CH_3_COOH (acetic acid), as described by Ozgen et al. [23]. After centrifuging the root and leaf samples at 12,298× *g* for 8 min at room temperature, 0.03 mL of supernatant was separated in a new centrifuge tube. This was followed by the addition of 2.97 mL of DPPH (0.1 mM) and incubation in the dark at room temperature for 30 min, as defined by Ozgen et al. [23]. The absorbance of the root and leaf samples was noted at 517 nm using a UV-1800 spectrophotometer (Shimadzu, Tokoyo, Japan). A standard curve using different concentrations of C_14_H_18_O_4_ Trolox (Sigma-Aldrich) was generated to estimate the DPPH capacity in mM Trolox/100 milligrams. Trolox is a water-soluble and cell-permeable compound with potent antioxidant properties, which is used for assessing the Trolox equivalent antioxidant capacity [23]. Furthermore, the DPPH free radical scavenging antioxidant activity (%) was calculated using the following formula:

Percentage of DPPH free radical scavenging activity (%) = 100 × [1 − {sample absorbance/control absorbance}].

### 2.4. Hydrogen Peroxide (H_2_O_2_) and Malondialdehyde (MDA) Contents

Citrus leaf tissues (0.1 mg) were ground in 1 mL of 1% C_2_HCl_3_O_2_ (trichloroacetic acid), while being kept on ice. The mixture was then spin in a centrifuge at 12,298× *g* for 10 min, at room temperature. Meanwhile, the potassium phosphate buffer pH 7 was prepared by mixing KH_2_PO_4_ (potassium dihydrogen phosphate) and K_2_HPO_4_ (dipotassium hydrogen phosphate). After centrifugation, 0.5 mL of the supernatant was combined with 0.5 mL of a 10 mM potassium phosphate buffer in a new tube. Subsequently, 1 mL of 1 M KI (potassium iodide) pH 7 was added to the tube. To determine the concentration of hydrogen peroxide, the absorbance of the reaction mixture was measured at 390 nm, using a spectrophotometer (UV-1800, Shimadzu). For the control, the aforementioned H_2_O_2_ procedure was repeated without the leaf samples. A standard curve was produced using commercial H_2_O_2_ (0 to 100 µmol) [24,25] and the results were expressed in micromoles of H_2_O_2_ per gram (µmol/g) of fresh weight [25].

The malondialdehyde levels were measured in terms of 0.1 mg of the leaves, by using a kit from the Nanjing Jiancheng Bioengineering Institute (kit A003-1). The MDA levels were determined according to the instructions provided by the producer of the kit.

### 2.5. Electrolytic Leakage and Reactive Oxygen Species Measurement

The Ohaus Starter 3100C apparatus (OHAUS Instruments, Shanghai, China) was utilized for the electrolytic leakage measurement. Initially, the leaves were cut into 1 × 1 cm small fragments and soaked in deionized water for 20 min, using a shaker, operating at 200 rounds per minute, at room temperature. Subsequently, the amount of electrolytic leakage was measured. Following this step, the sample solution underwent boiling for 15 min and the electrolytic leakage was measured again. The percentage of electrolytic leakage was then determined as outlined in reference [26]. The levels of reactive oxygen species were measured in 0.1 g of plant tissues, by using a kit (Elabscience #: EBC K138 F), and the amount of ROS was calculated based on the manufacturer’s instructions (https://www.elabscience.com/; accessed on 8 January 2024). In regard to the kit, the C_21_H_21_N_3_ (dihydroethidium) compound was used in an ROS assay, which is mainly oxidized by superoxide anion-type reactive oxygen species in cells.

### 2.6. Total Flavonoid Contents

#### 2.6.1. Root and Leaf Extraction Procedure

The total flavonoid contents were determined by homogenizing 0.1 g of leaf and root powder in 5 mL of 80% CH_3_OH methanol (Sigma-Aldrich), following the method by Velioglu et al. [27]. Homogenized samples were left in an orbital shaker, operating at 200 rpm for 2 h, at room temperature, followed by centrifugation at 12,298× *g* for 10 min. The aforementioned process was repeated twice and the combined supernatants were used to estimate the flavonoid content.

#### 2.6.2. Total Flavonoid Content in Root and Leaf

For the estimation of the total flavonoids, 0.5 mL of the citrus leaf and root solution, prepared as outlined above, was placed in a fresh tube and 2.25 mL of distilled water was added. After gently shaking, 0.15 mL of 5% NaNO_2_ (sodium nitrite) was added and followed by 6 min of incubation [28]. Following this, 0.3 mL of 10% AlCl_3_·6H_2_O (aluminum chloride hexahydrate) was added and vortexed, then incubated for 5 min at room temperature. Subsequently, 1 mL of 1 M NaOH (sodium hydroxide) solution was added to the vortexed solution and thoroughly mixed by gentle inversions, followed by 2 min of incubation. A UV-1800 spectrophotometer (Shimadzu) was used to measure the absorbance at 510 nm [28]. A standard curve for rutin (0–100 mg) was established to determine the total flavonoid content in milligrams of rutin per gram of root and leaf materials (mg rutin/g).

### 2.7. Chlorophyll and Relative Water Contents

Leaf powder (0.5 g) was placed in a centrifuge tube, followed by adding 10 mL of 80% C_3_H_6_O (acetone), and mixed well for 2 min [29]. The solution was incubated for 4 h in the dark at room temperature, than centrifuged for 10 min at 12,298× *g*. Repeat this procedure and combine the supernatant. A UV-1800 spectrophotometer (Shimadzu) was used to measure the absorbance of the supernatants at 645 nm and 663 nm, and the total chlorophyll was estimated, as described by Sumanta et al. [29].

Fresh leaves were collected for the relative water content (RWC) and the following formula was used:

RWC (%) = [(Fresh weight − dry weight)/(Turgid weight − dry weight)] × 100.

### 2.8. Statistical Analysis

Statistix 8.1 (Tallahassee, FL, USA) was utilized for the statistical analysis, presenting average values derived from three separate trials. The Excel program was used for the standard error estimation and the physiological and biochemical parameter graphs (Microsoft Corp., Redmond, WA, USA). Multiple statistical analysis techniques were used to group together individual compounds found in the flavonoid data related to plants under drought stress. The goal was to identify compounds that were very different from each other across different categories, while also being similar to each other within the same category. For the hierarchical cluster analysis (HCA) and principal component analysis (PCA), the concentration of each flavonoid compound was normalized by using R software (https://www.r-project.org/ accessed on 15 February 2024). Venn intersection analyses and interactive network studies of the root and leaf flavonoids were performed by using the EVenn program (free online tool) (http://www.ehbio.com/test/venn/#/ accessed on 15 May 2024).

## 3. Results

In this study, UPLC-MS/MS analysis was performed to identify the flavonoid profiles present in the root and leaf tissues of sour orange and lemon citrus species under drought stress. The study cataloged a total of 85 distinct flavonoids; their chemical formula, molecular weight, ion mode, parent and daughter ions, and other details, are provided in Supplementary Table S2. The flavonoids were classified into various subcategories, based on their chemical structure and properties. Specifically, the study identified 19 flavones and 19 flavonols, which are known for their antioxidant activities. Additionally, the analysis revealed the presence of 10 flavanones and 10 isoflavones, 9 chalcones, 6 flavanols, and 4 flavone glycosides, each contributing to the plant’s resilience and adaptive response to environmental stresses. Moreover, the study identified two flavanonols, two other flavonoid types, and one each of anthocyanin, chalcone glycoside, phenolic acids, and xanthones (Table S2). Each of these compounds plays a significant role in the plant’s physiological processes, particularly under stress conditions like drought, which can induce the synthesis of these secondary metabolites.

### 3.1. Symptoms of Drought Stress on Citrus Leaves and Roots

**Figure 1:**
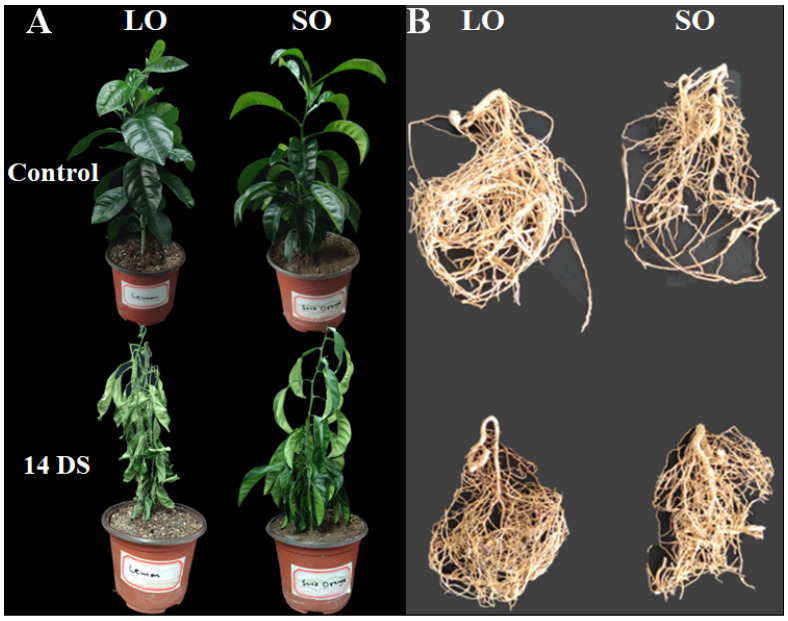
Symptoms of drought stress in citrus species. (**A**) Control plants (without stress) and 14 days of drought stress in lemon and sour orange plants showing drought symptoms on the leaves; (**B**) control roots (without stress) and drought stress symptoms on the LO and SO roots. Abbreviations: 14 DS: 14 days of drought stress, LO: lemon, SO: sour orange.

The lemon and sour orange species showed distinct phenotypes in the leaves and roots when exposed to drought stress. After 14 DS, sour orange (SO) leaves showed significant drought symptoms, such as leaf wilting and slight chlorosis (Figure 1A). After 14 DS, the lemon leaves showed extreme chlorosis and wrinkled phenotypes, as compared to control lemon leaves (Figure 1A). These leaf phenotypes showed that the lemon leaves were severely or permanently damage by the 14 DS and showed their susceptibility to drought, whereas the SO leaves predominantly tolerated the drought stress as compared to the lemon samples (Figure 1A).

Drought stress had detrimental effects on the lemon roots by reducing root growth compared to the control lemon roots (Figure 1B). After 14 DS, the lemon roots showed stunted root growth, root browning, root curling or twisting, and excessive dehydration of the root, showing the presence of the root wilting phenotype (Figure 1B). The SO roots also showed the presence of the curling or twisting phenotype after drought stress; however, the SO root did not show the presence of obvious root browning or excessive dehydration phenotypes (Figure 1B). These results indicate that the root system was significantly affected by 14 DS in lemon as compared to SO roots (Figure 1B).

### 3.2. Clustering and Grouping of Flavonoids under Drought Stress in Citrus Species

**Figure 2:**
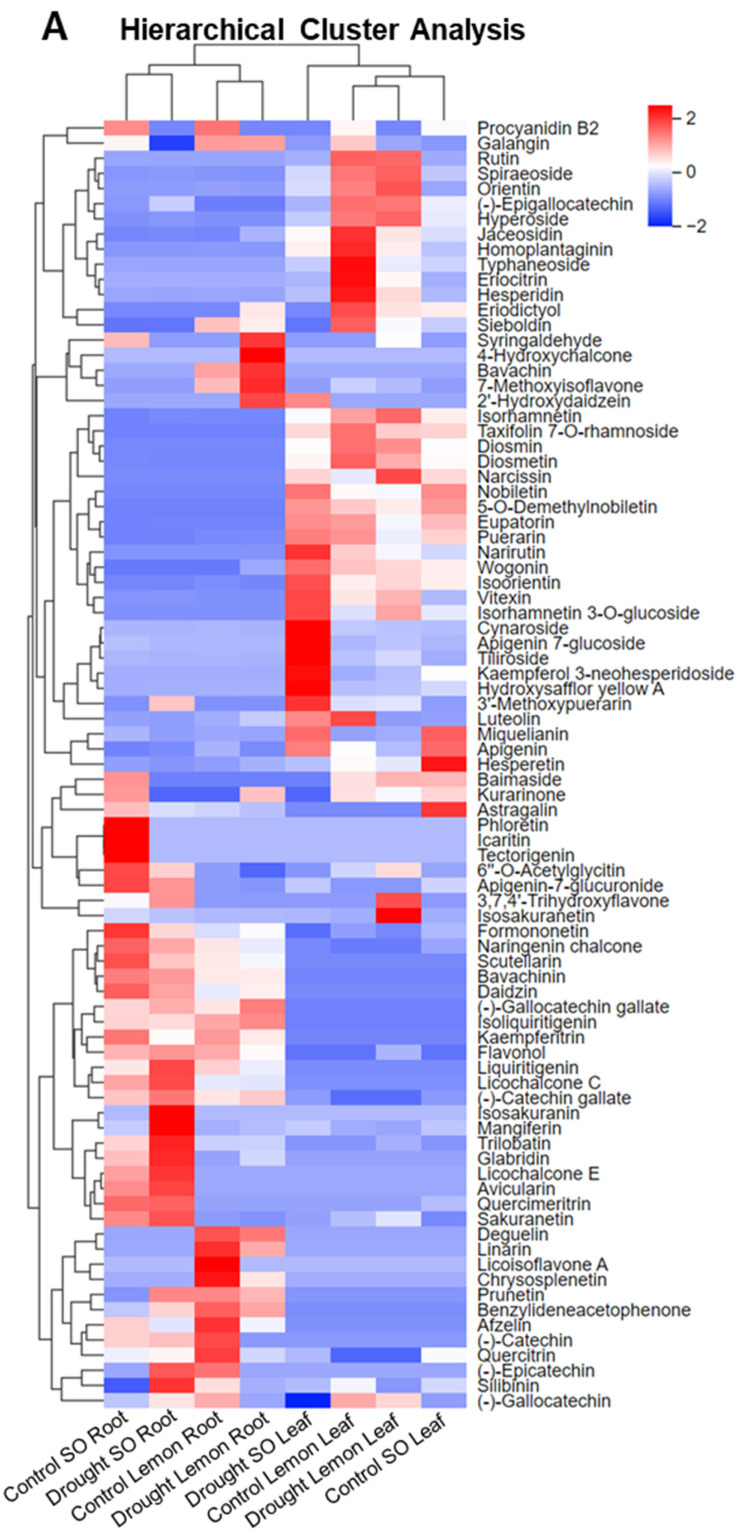

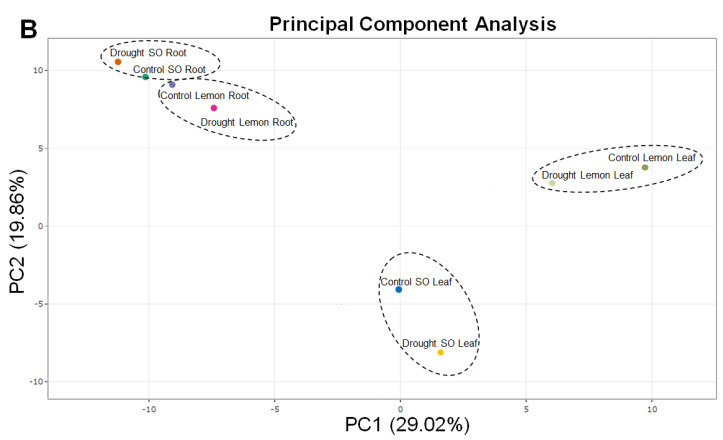
Citrus root and leaf flavonoid compound hierarchical cluster and principal component analyses, under drought stress. (**A**) Hierarchical cluster analysis of flavonoids among different tissues from citrus species, HCA columns show citrus samples and rows signify quantified flavonoids (rows are min/max normalized); (**B**) principal component analysis of different tissues from citrus species among the control and drought stress treatment samples. Each column/sample are the mean of three replicates. Abbreviations: SO: sour orange.

The heatmap illustrates the relative abundance of various flavonoid compounds in different sour orange and lemon samples, specifically focusing on the roots and leaves. Each row corresponds to a distinct flavonoid compound and each column represents different species subjected to drought stress treatment in terms of both root and leaf tissues. Notably, the column dendrograms reveal that SO and lemon root samples cluster separately. Specifically, SO roots exhibit a distinct flavonoid profile compared to that of lemon roots (Figure 2A). Post-drought stress, the flavonoid profile of SO leaves displays a unique pattern, not clustering with any other sample group and demonstrating a distinct abundance of flavonoid compounds, as shown in the hierarchical cluster analysis (Figure 2A).

Certain flavonoids, such as formononetin, naringenin chalcone, scutellarin, bavachinin, daidzin, (-)-gallocatechin gallate, isoliquiritigenin, kaempferitrin, liquiritigenin, licochalcone C, (-)-catechin gallate, and afzelin, exhibited elevated levels exclusively in all the root samples compared to the leaf samples, as illustrated in Figure 2. Conversely, flavonoids like isohamnetin, taxifolin 7-O-rhamnoside, diosmin, diosmetin, narcissin, nobiletin, wogonin, 5-O-demethylnobiletin, eupatorin, puerarin, narirutin, isoorientin, vitexin, and isorhamnetin 3-O-glucoside, were predominantly higher in terms of the amount in the leaf samples relative to the root samples (Figure 2A). This indicates that roots and leaves accumulate distinct types of flavonoid compounds to mitigate adverse drought stress conditions. These findings suggest that flavonoid accumulation varies among different species, with each flavonoid biosynthesizing unique compounds in response to drought stress. Furthermore, distinct flavonoid compounds are synthesized in leaves and roots.

In the drought-stressed SO roots, specific metabolites, such as isosakuranin, mangiferin, trilobatin, liquiritigenin, silibinin, avicularin, and glabridin, were found in notably higher amounts than in the control sour orange roots and drought-stressed lemon roots (Figure 2A). Additionally, drought-stressed lemon roots demonstrated a higher concentration of syringaldehyde, 4-hydroxychalcone, bavachin, 7-methoxyisoflavone, and 2′-hydroxydaidzein, compared to the control lemon group (Figure 2). Moreover, hydroxysafflor yellow A, tiliroside, cynaroside, and apigenin 7-glucoside, were found in significantly higher amounts in drought-stressed sour orange leaves than in drought-stressed lemon leaves (Figure 2A). Interestingly, 3′-methoxypuerarin was found in higher amounts in both the leaf and root tissues of drought-stressed sour orange samples than in drought-stressed lemon leaf and root tissues (Figure 2). The heatmap analysis reveals distinct flavonoid profiles in sour orange and lemon roots and leaves under drought stress. Specific flavonoids are elevated in different plant parts, indicating unique adaptive responses to drought stress. Sour orange roots and leaves show drought tolerance and unique flavonoid patterns compared to lemon roots and leaves, suggesting species-specific drought stress tolerance responses.

Principal component analysis was employed to investigate the variation in flavonoid compound abundance across different species and drought stress treatment groups. The first principal component (PC1) accounted for 29.02% of the total variance, while the second principal component (PC2) explained 19.86%, as depicted in Figure 2B. The distribution along the PC1 axis indicates a notable differentiation in the flavonoid profiles between the samples positioned on the right side (drought-stressed lemon leaf, control lemon leaf) and those on the left side (control SO root, drought-stressed SO root, control lemon root, drought-stressed lemon root). Additionally, the separation along the PC2 axis clearly distinguishes the flavonoid profiles of sour orange leaves from those of lemon roots, sour orange roots, and lemon leaves. The PCA plot reveals distinct clustering patterns among the species and drought stress treatment groups in both roots and leaves, highlighting variations in the flavonoid compound profiles.

In the PCA plot, the control SO root and drought-stressed SO root samples are closely clustered in the upper-left quadrant, indicating similar flavonoid compound profiles, with minimal variance between these two conditions. Below this cluster, the control lemon root and drought-stressed lemon root samples are grouped together, suggesting a different flavonoid compound profile compared to the sour orange group, as shown in Figure 2B. The control lemon leaf and drought-stressed lemon leaf samples are distinctly clustered in the right quadrant of the PCA plot, separate from the other groups, indicating a unique flavonoid compound profile in lemon leaves. Conversely, the control SO leaf and drought-stressed SO leaf samples are clustered together in the lower-left quadrant of the PCA plot, demonstrating distinct flavonoid compound abundance in sour orange leaves compared to the other groups (Figure 2B). These observations underscore that the flavonoid compound abundance varies not only between the drought stress and control treatments, but also between the leaf and root samples.

### 3.3. Venn Intersection and Interactive Network Analyses of Flavonoids

**Figure 3:**
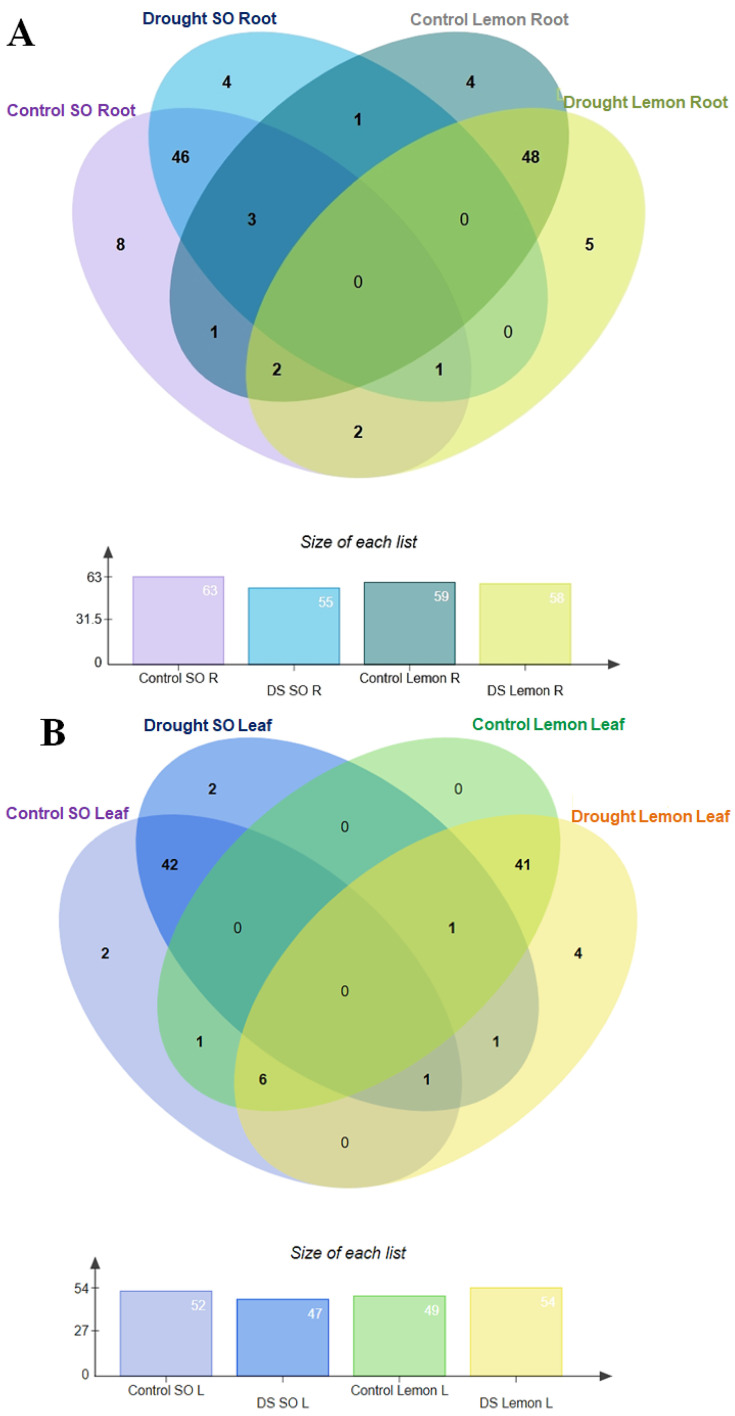
Venn intersection analyses of sour orange and lemon flavonoids. (**A**) Venn intersection analyses of sour orange and lemon root flavonoids; (**B**) Venn intersection analyses of sour orange and lemon leaf flavonoids. Abbreviations: R: root, L: leaf, DS: drought stress, SO: sour orange.

Venn intersection analyses of 85 quantified flavonoid compounds were performed to evaluate the variable impact of drought stress on flavonoid biosynthesis in different citrus species and tissues (Figure 3A,B). The analyses revealed differential distribution of these compounds across control and drought conditions and plant parts. Specifically, under control conditions, the root samples of SO exhibited the presence of 63 flavonoid compounds, while the roots of lemon plants contained 59 compounds (Figure 3A). Conversely, when subjected to drought stress, the flavonoid profile in SO roots showed a decrease to 55 compounds and lemon roots displayed a slight reduction to 58 compounds (Figure 3A).

Further exploration of the leaf samples under similar conditions provided additional insights (Figure 3B). The control samples of SO leaves exhibited 52 flavonoids, whereas the lemon leaves had 49 flavonoids (Figure 3B). Upon exposure to drought stress, a notable alteration in the flavonoid composition was observed; SO leaves demonstrated a reduction to 47 flavonoids, whereas lemon leaves exhibited an increase to 54 flavonoids (Figure 3B). These findings, as shown in Figure 3A,B, underscore the variable impact of environmental stressors on the flavonoid biosynthesis pathways in different citrus species and tissues. This differential response highlights the complex metabolic adjustments plants undergo in response to drought stress, reflecting their adaptive mechanisms at the metabolic level.

**Figure 4:**
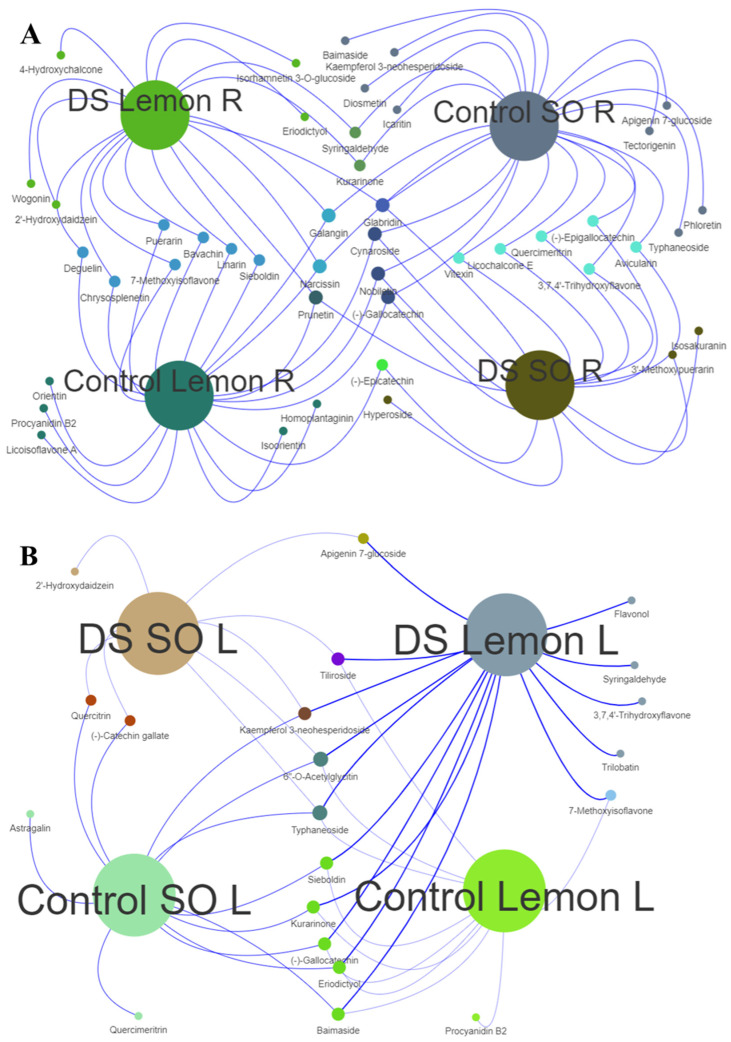
Venn interactive networks of sour orange and lemon flavonoids. (**A**) Venn interactive network of sour orange and lemon root flavonoids; (**B**) Venn interactive network of sour orange and lemon leaf flavonoids. Abbreviations: R: root, L: leaf, DS: drought stress, SO: sour orange.

Venn interactive analyses were performed to identify the flavonoid profiles in the root and leaves of the sour orange and lemon species subjected to both control and drought stress environments (Figure 4A,B). The control lemon roots exhibited a distinctive profile in terms of flavonoids, such as orientin, procyanidin B2, licoisoflavone A, isoorientin, and homoplantaginin; however, after drought stress, these specific compounds were no longer detectable in the lemon root samples (Figure 4A). This indicates a significant alteration in the biosynthetic pathway of flavonoids due to the stress condition. Conversely, after drought stress a unique set of flavonoids were identified in the lemon roots, such as 4-hydroxychalcone, isorhamnetin 3-O-glucoside, eriodictyol, wogonin, and 2′-hydroxydaidzein, suggesting an adaptive metabolic response to counteract the stress effects (Figure 4A). Similarly, the control SO root samples demonstrated a unique flavonoid composition, such as typhaneoside, baimaside, icaritin, kaempferol 3-neohesperidoside, tectorigenin, diosmetin, phloretin, and apigenin 7-glucoside (Figure 4A). Following the application of drought stress, there was a notable shift in the SO root flavonoids, such as hyperoside, 3-methoxypuerarin, and isosakuranin (maybe these flavonoids are more protective under drought conditions), which are not typically identified under control conditions (Figure 4A). This shift could be indicative of a stress-induced modulation of flavonoid biosynthesis, potentially contributing to enhanced stress tolerance.

Apigenin 7-glucoside was detected not only in drought-stressed sour orange leaves, but also in lemon leaves, indicating a common adaptive response to water scarcity across these citrus species (Figure 4B). After DS, the SO leaves revealed the presence of 2′-hydroxydaidzein (Figure 4B). Several flavonoids uniquely found in lemon leaves under drought stress, such as flavonol, syringaldehyde, 3,7,4′-trihydroxyflavone, and trilobatin, were not detected in the control plants (Figure 4B). This suggests a specific induction of these compounds as a protective mechanism against oxidative stress associated with drought conditions. In contrast, the leaves of the control SO samples exhibited the presence of astragalin and quercimeritrin, while both the control and drought-stressed leaves of this species consistently showed the presence of quercitrin and (-)-catechin gallate (Figure 4B). This pattern suggests a baseline level of these flavonoids in sour orange, which may play a role in the plant’s inherent resilience to drought stress. Moreover, the study also noted the occurrence of 7-methoxyisoflavone in both lemon leaves and roots under control and drought conditions, pointing to the possibility that certain flavonoids may be species-specific and could potentially serve as biomarkers for drought tolerance in citrus plants (Figure 4). The consistent presence of 7-methoxyisoflavone across different tissues and environmental conditions underscores its potential role in the plant’s overall defensive strategies against drought stress.

### 3.4. Flavonoid Fold Change in Citrus Species under Drought Stress

Under drought stress, the root and leaf tissues of sour orange and lemon species exhibited significant alterations in the flavonoid compounds present, with changes exceeding the 2-fold mark (Table 1). In the root tissues of SO, several compounds, including benzylideneacetophenone, trilobatin, (-)-epigallocatechin, liquiritigenin, silibinin, 3,7,4′-trihydroxyflavone, and isorhamnetin, were increased by more than 2-fold under drought stress compared to control SO roots (Table 1). These compounds may play a crucial role in mitigating the adverse effects of drought stress in SO roots. Conversely, in lemon roots, only two flavonoids, hesperetin and jaceosidin, were increased by 2.71- and 4.11-fold, respectively (Table 1). Additionally, hesperetin levels were decreased by −3.92-fold in SO roots (Table 1). This indicates the species-specific regulation of flavonoids under similar drought conditions. In both SO and lemon species, hesperidin levels were decreased by −9.22- and −10.94-fold, respectively (Table 1), which suggests a strategic metabolic shift aimed at optimizing the plant’s defense mechanisms.

In the case of sour orange leaves, 14 distinct flavonoids exhibited more than a 2-fold upregulation under drought conditions. Notably, cynaroside and 3′-methoxypuerarin demonstrated significant increases, with levels rising by 26.15- and 24.86-fold, respectively (Table 1). These results highlight the unique adaptive mechanisms employed by sour orange leaves in response to drought stress. In contrast, lemon leaves displayed a divergent pattern in terms of flavonoid regulation under similar drought conditions. Specifically, 10 flavonoids were downregulated by more than 2-fold, indicating a substantial reduction in their biosynthesis or accumulation. However, four flavonoids, namely isosakuranetin, narcissin, tiliroside, and isorhamnetin 3-O-glucoside, were upregulated by more than 2-fold (Table 1).

### 3.5. Flavonoids Biosynthesis Gene Expression Profiling

**Figure 5:**
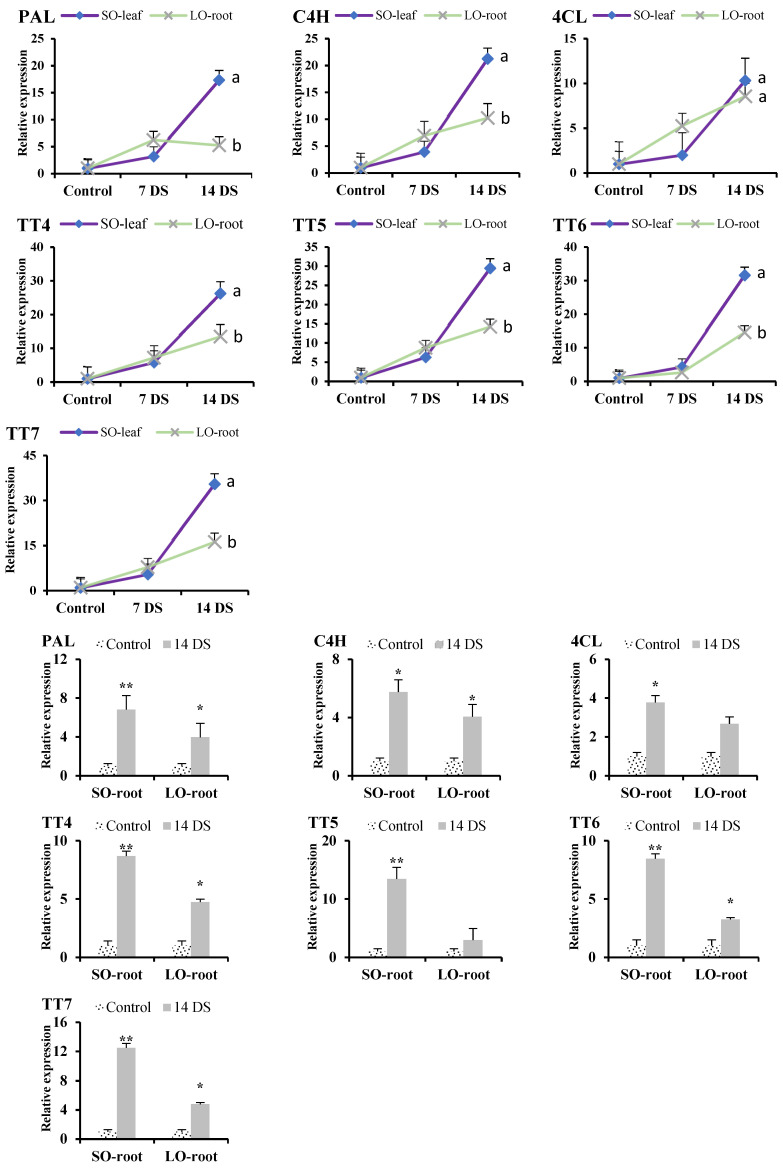
Expression analysis of citrus root and leaf flavonoid biosynthesis genes under drought stress. Abbreviations: SO: sour orange (SO), LO: lemon, PAL: phenylalanine ammonia-lyase; C4H: cinnamate 4-hydroxylase, 4CL: 4-coumarate: coenzyme A ligase, TT4: transparent testa 4, TT5: transparent testa 5, TT6: transparent testa 6, TT7: transparent testa 7. Each figure represents the mean of 3 replications and the bars signify the standard error. The least significant difference (LSD) was used at *p* < 0.05 (a,b), whereas for the root data, Student’s t-test was used to compare the control and drought treatment at * *p* < 0.05 and ** *p* < 0.01.

In this study, we conducted a comprehensive analysis of the expression profiles of seven key genes involved in the flavonoid biosynthesis pathway. These genes were examined in both the leaves and roots of lemon and SO subjected to drought stress conditions. Initially, at 7 DS, the expression levels of PAL, C4H, 4CL, TT4, and TT7 were assessed (Figure 5). The results indicated a slight elevation in the expression of these genes in lemon leaves compared to SO leaves. However, this increase was not statistically significant, suggesting that the early response to drought stress may not obviously differ between these two citrus species in terms of flavonoid biosynthesis gene expression. The analysis conducted after 14 DS revealed a more pronounced change in the gene expression. In SO leaves, there was a significant upregulation in the expression of PAL, C4H, TT4, TT5, TT6, and TT7, compared to lemon leaves (Figure 5). This significant enhancement suggests that the flavonoid biosynthesis pathway is more actively engaged in SO leaves under prolonged drought conditions. The increased production of flavonoid compounds in SO leaves likely serves as a protective mechanism against oxidative stress induced by the accumulation of ROS, which are commonly elevated during abiotic stress conditions, such as drought.

In the roots of SO, there was a significant upregulation in the expression of all seven studied genes (*PAL*, *C4H*, *4CL*, *TT4*, *TT5*, *TT6*, and *TT7*) following drought stress. This indicates a robust activation of the flavonoid biosynthesis pathway, potentially contributing to the enhanced stress tolerance observed in SO roots. In contrast, lemon roots displayed increased expression levels of 4CL and TT5 post-drought stress; however, these increases did not reach statistical significance, suggesting a less pronounced activation of the flavonoid biosynthesis pathway in lemon roots under similar stress conditions. These findings underscore the differential activation of the flavonoid biosynthesis pathway between lemon and sour orange in response to drought stress, highlighting potential species-specific adaptations to environmental stressors. The results contribute valuable insights into the molecular mechanisms underlying citrus responses to drought and suggest that manipulating flavonoid biosynthesis pathways could be a viable strategy for enhancing drought tolerance in citrus species.

### 3.6. Flavonoid Content and Antioxidant Activity under Drought Stress

**Figure 6:**
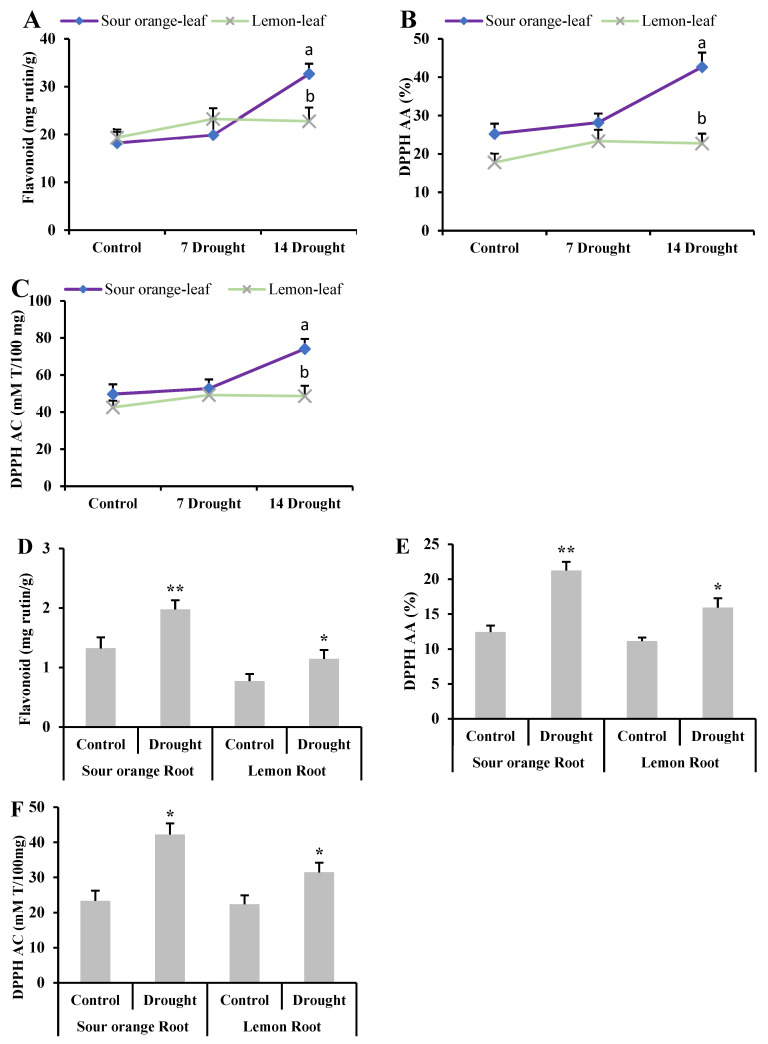
Flavonoid content and DPPH activity and capacity in citrus leaf and root samples under drought stress. (**A**) Total flavonoid content in leaf samples, (**B**) leaf DPPH antioxidant activity, (**C**) leaf DPPH antioxidant capacity, (**D**) root total flavonoid content, (**E**) root DPPH antioxidant activity (%), (**F**) root DPPH antioxidant capacity. Abbreviations: DPPH: 2,2-diphenyl-1-picrylhydrazyl; AA: antioxidant activity; AC: antioxidant capacity; 7 Drought: 7 days of drought stress; 14 Drought: 14 days of drought stress. Each figure represents the mean of 3 replications and the bars signify the standard error. The least significant difference (LSD) was used at *p* < 0.05 (a,b), whereas for the root data, Student’s t-test was used at * *p* < 0.05 and ** *p* < 0.01.

After 7 DS, the flavonoid concentration in lemon leaves was 23.26 mg rutin/g, while in SO leaves, it was slightly lower at 19.94 mg rutin/g (Figure 6). This initial observation suggested a modest increase in the lemon samples, which was not significant. However, a pronounced change was observed after 14 DS. At this stage, the sour orange leaves exhibited a substantial increase in the total flavonoid content, reaching 32.68 mg rutin/g, compared to lemon leaves, which was only 22.78 mg rutin/g (Figure 6). This obvious difference in flavonoid accumulation between the two species under prolonged drought conditions indicates that sour orange is considerably more efficient at upregulating flavonoid biosynthesis in response to extended periods of water deficit. In contrast, lemon leaves were less capable of enhancing flavonoid synthesis to mitigate the negative effects of prolonged drought stress.

Further analysis revealed that the antioxidant properties of the leaves also differed significantly between the two species after 14 days of drought stress. Specifically, sour orange leaves demonstrated a higher level of antioxidant activity 42.68% and a greater antioxidant capacity 74.14 mM Trolox/100 mg (Figure 6). In comparison, lemon leaves showed lower antioxidant activity and capacity, at 22.76% and 48.65 mM Trolox/100 mg, respectively (Figure 6). These findings underscore the superior physiological adaptation of sour orange leaves in terms of both flavonoid accumulation and antioxidant defense mechanisms under drought stress conditions.

In the case of root tissues subjected to the same 14-day drought stress period, similar trends were observed. The roots of sour orange plants contained significantly higher levels of flavonoids, at 1.97 mg rutin/g, compared to 1.14 mg rutin/g in lemon roots (Figure 6). Additionally, the antioxidant activity and capacity in sour orange roots were measured at 21.25% and 42.23 mM Trolox/100 mg, respectively, surpassing the 15.93% and 31.45 mM Trolox/100 mg recorded in lemon roots (Figure 6). The comparative analyses of both leaf and root tissues highlight the robustness of sour orange in deploying secondary metabolic pathways that enhance the flavonoid content and antioxidant properties, thereby conferring an improved adaptive response to drought stress compared to the lemon samples.

### 3.7. Physiological and Biochemical Responses of Citrus Species under Drought Stress

**Figure 7:**
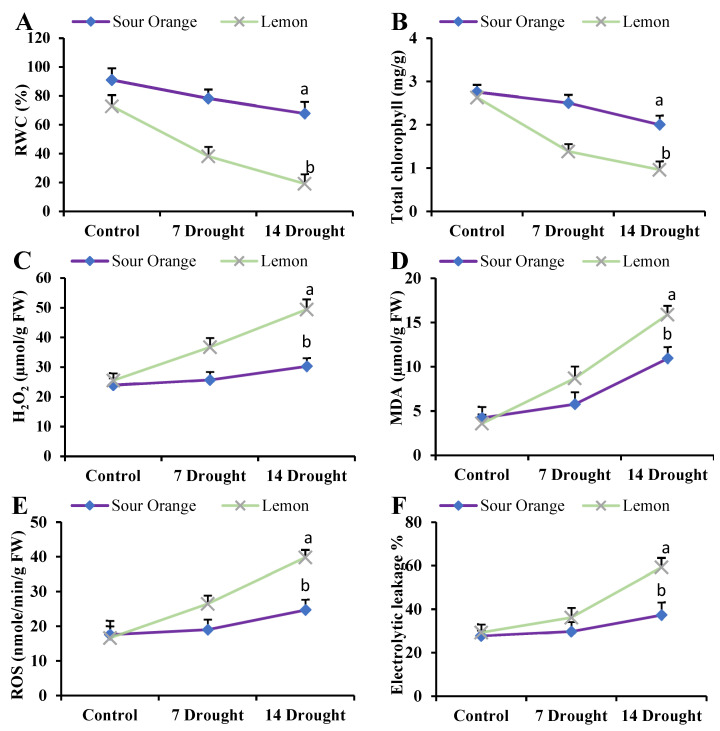
Drought stress effects on citrus leaf biochemical parameters. (**A**) Relative water content, (**B**) total chlorophyll content, (**C**) H_2_O_2_ content, (**D**) malondialdehyde content, (**E**) reactive oxygen species, (**F**) electrolytic leakage. Abbreviations: RWC: relative water content; H_2_O_2_: hydrogen peroxide; MDA: malondialdehyde; ROS: reactive oxygen species; FW: fresh weight; 7 Drought: 7 days of drought stress; 14 Drought: 14 days of drought stress. Each figure represents the mean of 3 replications and the bars signify the standard error. The least significant difference (LSD) was used at *p* < 0.05 (a,b).

We also investigated the physiological responses of sour orange and lemon species to drought stress, focusing on several key biochemical parameters, such as the relative water content, chlorophyll content, H_2_O_2_, malondialdehyde, ROS, and electrolytic leakage (EL), which are indicative of stress tolerance. The results demonstrated a significant difference in the drought tolerance between the two species. Sour orange leaves exhibited a considerably higher RWC of 67.93% compared to 19.19% in lemon leaves, after 14 days of drought stress, suggesting a better water retention capability (Figure 7A).

Additionally, the total chlorophyll content was significantly higher at 2.006 mg/g of fresh weight (FW) in sour orange, as compared to lemon leaves 0.963 mg/g FW (Figure 7B). These findings indicate more robust photosynthetic machinery in the sour orange samples under drought stress, which could be attributed to better water retention and possibly other adaptive physiological mechanisms. On the other hand, the oxidative stress markers showed a contrasting pattern. The levels of H_2_O_2_, MDA, ROS, and EL were significantly lower in sour orange leaves, with values of 30.37 µmol/g FW, 10.96 µmol/g FW, 24.75 nmole/min/g FW, and 47.46%, respectively (Figure 7C–F). In contrast, lemon leaves demonstrated higher levels of these stress markers, with H_2_O_2_ of 49.45 µmol/g FW, MDA of 15.89 µmol/g FW, ROS of 39.82 nmole/min/g FW, and EL of 69.26% (Figure 7C–F). These results suggest that lemon leaves are less efficient at mitigating oxidative damage induced by drought stress, which correlates with their lower RWC and chlorophyll content.

The comparative analysis of these physiological and biochemical parameters clearly illustrates that sour orange leaves and roots are more adept at alleviating drought-induced stress. The lower levels of oxidative stress markers in sour orange, coupled with higher antioxidant capacity, chlorophyll content, and enhanced flavonoid content, point towards a comprehensive set of adaptive responses that confer enhanced drought tolerance. This resilience in sour orange could be attributed to its ability to effectively modulate physiological and biochemical pathways to mitigate the adverse effects of prolonged drought conditions, thereby maintaining cellular integrity and function. In contrast, the lemon species exhibits a reduced capacity to cope with similar stress conditions, as evidenced by its higher oxidative stress levels and lower chlorophyll content, which ultimately compromise its physiological functionality under prolonged drought stress.

## 4. Discussion

The Citrus genus comprises over 162 species, each characterized by unique and varied levels of secondary metabolites [10,30]. These metabolites serve as a secondary defense mechanism, aiding in the normal growth, development, and environmental interactions of the plants. Recent research has shown that certain citrus species, such as *Carrizo citrange* and *Citrus latipes*, exhibit high levels of these metabolites, enabling them to more effectively neutralize ROS during both abiotic and biotic stresses [7,31]. Typically, commercially grown citrus types, like lemons, pummelos, sweet oranges, and mandarins, are more vulnerable to ROS damage incurred during stress, as these varieties have lower metabolite levels [7,32]. Our findings demonstrate that drought-resistant sour orange leaves and roots showed significantly higher levels of flavonoid compounds, antioxidant activities, and a lower level of reactive oxygen species under drought conditions, compared to the drought-sensitive lemon leaves and root tissues (Figure 2A,B, Figure 6, and Figure 7).

In citrus species, the synthesis of flavonoids has been increasingly triggered by various biotic and abiotic stresses (including metal toxicity, drought, injury, high light intensity, cold, salt stress, harmful radiation absorption, and nutrient shortages) [33]. In transgenic Arabidopsis, the overexpression of the citrus *CsCYT75B1* gene enhances the activity of flavonoid biosynthesis genes like *PAL*, *C4H*, *4CL*, *TT4*, *TT5*, *TT6*, and TT7 under drought conditions, leading to higher total flavonoid level compared to the wild type (WT). These transgenic lines better adapt to drought stress by neutralizing ROS more effectively than the WT [34]. Our qPCR analysis indicates that the leaf and root tissues of sour orange exhibit significantly greater expression of the genes *PAL*, *C4H*, *TT4*, *TT5*, *TT6*, and *TT7* compared to lemon, after 14 DS (Figure 5). This differential expression of flavonoid biosynthesis genes in both citrus species under drought conditions suggests species-specific regulation that could be attributed to genetic differences, which influenced the expression of key enzymes involved in flavonoid biosynthesis. Previous literature has noted that genes involved in flavonoid biosynthesis, such as *PAL*, *C4H*, *4CL*, *TT4*, *TT5*, *TT6*, and *TT7*, are notably active in citrus species during drought stress. The activation of these genes promotes the production of antioxidant flavonoids, like flavanone, flavones, and flavonols [7,18]. These outcomes suggest that the biosynthesis of flavonoids is crucial in reducing the adverse effects of drought stress and aiding the adaptation of sour orange to semi-arid conditions.

In drought-stressed sour orange roots, a specific array of flavonoids, including isosakuranin, mangiferin, trilobatin, liquiritigenin, silibinin, and glabridin, exhibited significantly elevated levels compared to the roots and leaves of lemon under 14 DS (Figure 2). Moreover, the flavonoid profiles of control sour orange root samples showed the presence of various flavonoids, including typhaneoside, baimaside, icaritin, kaempferol 3-neohesperidoside, tectorigenin, diosmetin, phloretin, and apigenin 7-glucoside (Figure 4A). Prior studies have highlighted that the aforementioned flavonoids possess significant antioxidant properties and offer various health benefits, including anticancer, anti-inflammatory, antidiabetic, gastroprotective, antidepressant, cardioprotective, antifungal, radioprotective, and neuroprotective effects [17,35]. Additionally, scion metabolites are influenced by rootstock metabolites; rootstocks with high levels of phenolics, flavonoids, and alkaloids enhance the metabolic levels of grafted scions [36]. A recent study showed that rootstocks significantly enhanced the secondary metabolites in grafted citrus fruits [37], and also improved the anthocyanin content by 63.6% and the antioxidant activities by 48.92% in *Citrus sinensis* blood orange Tarocco Sciré [38]. Therefore, rootstocks with high flavonoid levels can be selected for grafting to improve the flavonoid levels in scions, thereby producing fruits with enhanced antioxidant properties.

In sour orange root tissues, isosakuranin, hyperoside, and 3-methoxypuerarin were identified, while in the leaf tissues, the synthesis of 2′-hydroxydaidzein, quercitrin, and (-)-catechin gallate was observed (Figure 4A,B). In contrast, lemon plants did not show the presence of these flavonoids in the root or leaf tissues after 14 days of drought stress (Figure 4A,B). These flavonoids potentially contribute to effectively scavenging ROS [31,35,39,40,41] and enhanced sour orange’s resilience against drought stress compared to lemon plants. After drought stress, a significant alteration in the flavonoids was observed. Notably, flavonoids such as 3-methoxypuerarin, isosakuranin, and hyperoside were detected, which are typically absent under normal watering conditions, suggesting the stress-induced modulation of flavonoid biosynthesis. These findings align with previous studies indicating that phenolics and flavonoid biosynthesis are stimulated in sweet basil (*Ocimum basilicum* L.) and *Anoectochilus roxburghii* (Wall.) Lindl. under oxidative stress caused by ultraviolet-B irradiation and heat stress [42,43]. Furthermore, they underscore the potential utility of specific flavonoids as biomarkers for monitoring and potentially improving stress tolerance in plants. The disappearance of certain flavonoids and the emergence of new ones in response to drought stress may reflect an intrinsic biochemical strategy of plants to mitigate the adverse effects of water scarcity. These findings contribute to our understanding of the complex metabolic adjustments that citrus plants undergo in response to drought stress and highlight the potential of specific flavonoids as indicators of stress adaptation.

Under heat stress, the isorhamnetin contents were increased by 5.01-fold in *Anoectochilus roxburghii* (Wall.) Lindl.; however, rutin was decreased in response to heat stress [43]. Our results showed that isorhamnetin was increased by 2.43-fold in sour orange roots, whereas rutin was decreased by −2.17-fold and −2.14-fold in root tissues of sour orange and lemon, respectively. Isorhamnetin protects against oxidative stress by activating Nrf2 and inducing the expression of its target genes [44]. Benzylideneacetophenone was significantly increased by 15-fold in sea buckthorn (*Hippophae rhamnoides* L.) under drying stress [45]. Our results showed that the root tissues of sour orange were increased in benzylideneacetophenone by 2.59-fold as compared to the control sour orange roots (Table 1). Under drought stress, licorice (*Glycyrrhiza Tourn.* L.) actively synthesized the liquiritigenin bioactive compound [46]. Liquiritigenin was increased by 2.01-fold in sour orange roots under drought stress compared to the control roots (Table 1).

The tea plant (*Camellia sinensis*) accumulates significant quantities of epigallocatechin and tolerates drought stress [47]. Trilobatin, a dihydrochalcone compound, can better protect plants by removing excessive ROS and filtering ultraviolet radiation [48]; whereas the silibinin compound is widely known for its antitumor activities [49]. Our results displayed an increase in trilobatin by 2.07-fold in sour orange roots and both (-)-epigallocatechin and silibinin were increased by 3.01-fold in sour orange roots (Table 1). Interestingly, the leaves of sour orange showed a decrease in (-)-epigallocatechin by −2.08-fold (Table 1). The (-)-epigallocatechin increase in the roots and decrease in the leaves of sour orange shows that the drought stress adaptive mechanisms also differ in different organs (leaves and roots) of the same plant. Our results and their comparison with previous studies leads to the conclusion that sour orange root and leaf tissues under drought increased the levels of flavonoids that are known for their antioxidant properties and which could be crucial in alleviating oxidative stress induced by drought conditions.

## 5. Conclusions

We conclude that the sour orange leaf and root tissues showed better performance than lemon leaf and root tissues under drought stress. Sour orange root tissues showed significantly high accumulation of antioxidant flavonoids, namely silibinin, benzylideneacetophenone, liquiritigenin, isorhamnetin, trilobatin, (-)-epigallocatechin, and 3,7,4′-trihydroxyflavone, by more than 2-fold under drought stress compared to the control sour orange roots. However, in lemon roots only jaceosidin and hesperetin were increased by 4.11- and 2.71-fold, respectively, compared to control lemon roots. In the leaves of drought-stressed sour orange, flavonoids, like hydroxysafflor yellow A, cynaroside, tiliroside, and apigenin 7-glucoside, were found in higher amounts than in drought-stressed lemon leaves. Additionally, under drought stress, sour orange leaf and root tissues had higher expression in terms of flavonoid biosynthesis genes, flavonoid content, antioxidant activities, and relative water content, along with lower reactive oxygen species than lemon leaf and root tissues, contributing to a better understanding of plant resilience to drought stress.

## Figures and Tables

**Table 1:** Flavonoids with a more than 2-fold change in drought stress conditions in sour orange (SO) and lemon tissues (root and leaves).

Serial No.	Compounds	Class	Fold Change in Drought-Stressed SO Root	Fold Change in Drought-Stressed Lemon Root	Fold Change in Drought-Stressed SO Leaf	Fold Change in Drought-Stressed Lemon Leaf
1	Hydroxysafflor yellow A	-	-	-	6.51	-
2	Benzylideneacetophenone	Chalcones	2.59	-	-	-
3	Trilobatin	Chalcones	2.07	-	-	-
4	Sieboldin	Chalcones	-	-	-	−2.08
5	Naringenin chalcone	Chalcones	-	-	−2.80	-
6	(-)-Epigallocatechin	Flavanols	3.01	-	−2.08	-
7	(-)-Gallocatechin	Flavanols	-	−2.20	-	-
8	Isosakuranetin	Flavanones	-	-	9.18	26.34
9	Liquiritigenin	Biflavonoids	2.01	-	-	-
10	Hesperidin	Flavanones	−9.22	−10.94	-	−2.37
11	Hesperetin	Flavanones	−3.92	2.71	−6.76	-
12	Eriocitrin	Flavanones	-	-	3.59	−3.28
13	Eriodictyol	Flavanones	-	-	-	−1.92
14	Narirutin	Flavanones	-	-	4.08	-
15	Silibinin	Flavanonols	3.01	-	-	-
16	Isoorientin	Flavone glycosides	-	-	2.02	-
17	Vitexin	Flavone glycosides	-	-	6.42	-
18	3′-Methoxypuerarin	Flavone glycosides	-	-	24.86	-
19	Orientin	Flavone glycosides	-	-	5.67	-
20	Chrysosplenetin	Flavones	-	−2.75	-	-
21	Luteolin	Flavones	-	-	5.80	−7.91
22	Sakuranetin	Flavones	-	-	2.79	-
23	Jaceosidin	Flavones	-	4.11	-	−1.99
24	Diosmin	Flavones	−3.18	-	-	-
25	Apigenin	Flavones	-	−2.04	-	-
26	5-O-Demethylnobiletin	Flavones	-	−2.42	-	-
27	Homoplantaginin	Flavones	-	-	2.54	−2.42
28	Galangin	Flavones	-	-	-	−2.31
29	Narcissin	Flavones	-	-	-	2.91
30	Cynaroside	Flavones	-	-	26.15	-
31	Nobiletin	Flavones	−23.95	-	-	-
32	Astragalin	Flavonols	−2.08	-	-	-
33	Rutin	Flavonols	−2.17	−2.14	-	-
34	Tiliroside	Flavonols	-	-	-	2.01
35	Quercitrin	Flavonols	-	−2.85	-	-
36	3,7,4′-Trihydroxyflavone	Flavonols	2.05	-	-	-
37	Isorhamnetin 3-O-glucoside	Flavonols	-	-	3.03	2.36
38	Typhaneoside	Flavonols	-	-	-	−4.21
39	Kaempferol 3-neohesperidoside	Flavonols	-	-	3.49	-
40	Afzelin	Flavonols	-	−2.92	-	-
41	Isorhamnetin	Flavonols	2.43	-	-	-
42	6″-O-Acetylglycitin	Isoflavones	-	−2.71	-	-
43	Formononetin	Isoflavones	-	-	−7.65	-
44	Puerarin	Isoflavones	-	-	-	−2.00

## Data Availability

All the data are available in the main text.

## Supplementary Materials

The following are available online at https://www.mdpi.com/article/10.3390/antiox13091149/s1, File S1: Signifies the UPLC instrument analytical conditions and ESI-MS/MS parameters. Table S1: Retention times (RTs) and calibration curves for the standards and raw flavonoid concentration (nmol/g) in root and leaf tissues of sour orange and lemon. Table S2: Illustrate the 85 distinct flavonoids, their chemical formula, molecular weight, ion mode, parent and daughter ions, and other details.
