# Supplementary material for: Comparative Analysis of Citrus Species’ Flavonoid Metabolism, Gene Expression Profiling, and Their Antioxidant Capacity under Drought Stress
Source: Antioxidants (Basel). 2024 Sep 23;13(9):1149. doi: 10.3390/antiox13091149 (PMC11428974; doi:10.3390/antiox13091149)
Supplement: Supplementary file 1 [file antioxidants-13-01149-s001.zip › Supplementary File S1.pdf]

UPLC-ESI-MS/MS system conditions

The sample extracts were examined using a UPLC-ESI-MS/MS system (UPLC, ExionLC™ AD, https://sciex.com.cn/; MS, Applied Biosystems 6500 Triple Quadrupole, https://sciex.com.cn/). The analytical parameters were as follows: UPLC column, Waters ACQUITY UPLC HSS T3 C18 (100 mm×2.1 mm, 1.8 μm); solvent system, water with 0.05% formic acid (A), acetonitrile with 0.05% formic acid (B). The gradient elution program was set as follows: 0-1 min, 10%-20% B; 1-9 min, 20%-70% B; 9-12.5 min, 70%-95% B; 12.5-13.5 min, 95% B; 13.5-13.6 min, 95%-10% B; 13.6-15 min, 10% B. The flow rate was 0.35 mL/min, the temperature was maintained at 40°C, and the injection volume was 2 μL.

ESI-MS/MS operation parameters

Linear ion trap (LIT) and triple quadrupole (QQQ) scans were performed using a triple quadrupole-linear ion trap mass spectrometer (QTRAP), specifically the QTRAP® 6500+ LC-MS/MS System. This system was equipped with an ESI Turbo Ion-Spray interface, functioning in both positive and negative ion modes, and managed by Analyst 1.6.3 software (Sciex). The ESI source parameters were set as follows: ion source, ESI+/-; source temperature at 550 °C; ion spray voltage (IS) at 5500 V (Positive) and -4500 V (Negative); and curtain gas (CUR) at 35 psi. Flavonoids were analyzed using scheduled multiple reaction monitoring (MRM). Data acquisition was conducted using Analyst 1.6.3 software (Sciex), and Multiquant 3.0.3 software (Sciex) was utilized for metabolite quantification. The mass spectrometer parameters, including declustering potentials (DP) and collision energies (CE) for individual MRM transitions, were optimized further. Specific MRM transitions were monitored for each period based on the metabolites eluted during that time.

The qualitative analysis of 85 flavonoids was performed by comparing the accurate precursor ions (Q1), product ion (Q3) values, fragmentation patterns, and retention times (as shown in supplementary table 1) with those obtained from standard injections under identical conditions. The standards were sourced from Sigma-Aldrich, St. Louis, MO, USA (http://www.sigmaaldrich.com/united-states.html).

Flavonoid quantification formula

To determine the flavonoid content in citrus samples, the integral peak area ratio of each sample is substituted into the linear equation of the standard curve. This value is then used in the calculation formula to obtain the absolute content of the substance in the sample, as previously described (Chen et al., 2013). The formula has been converted into appropriate units, allowing for direct substitution of the sample size. The flavonoid content in the sample (nmol/g) is calculated using the formula: $C \times V / 1000000 / W$. Here, C represents the sample concentration value obtained from the standard curve (nmol/L), W is the weighed sample mass (g), and V is the solution volume used for extraction (μL).

Supplementary Table S2. Illustrate the 85 distinct flavonoids, their chemical formula, molecular weight, ion mode, parent and daughter ions, and other details.

Srial No.	Compounds	Class	Formula	CAS	KEGG ID	Molecular Weight	Q1 (Da)	Q3 (Da)	Ion mode
1	Hydroxysafflor yellow A	Chalcone glycoside	$C_{27}H_{32}O_{16}$	78281-02-4	-	612.2	611.2	491.1	-ive
2	Deguelin	Other flavonoids	$C_{23}H_{22}O_6$	522-17-8	C10417	394.1	395.2	213.1	+ive
3	Procyanidin B2	Anthocyanins	$C_{30}H_{26}O_{12}$	29106-49-8	C17639	578.1	577.1	407.1	-ive
4	Licochalcone E	Chalcones	$C_{21}H_{22}O_4$	864232-34-8	-	338.2	337.1	187.1	-ive
5	4-Hydroxychalcone	Chalcones	$C_{15}H_{12}O_2$	20426-12-4	C14231	224.1	223.1	117.1	-ive
6	Licochalcone C	Chalcones	$C_{21}H_{22}O_4$	144506-14-9	-	338.2	337.1	279.1	-ive
7	Benzylideneacetophenone	Chalcones	$C_{15}H_{12}O$	94-41-7	C01484	208.1	209.09	103.05	+ive
8	Trilobatin	Chalcones	$C_{21}H_{24}O_{10}$	4192-90-9	-	436.1	435.13	273.08	-ive
9	Isoliquiritigenin	Chalcones	$C_{15}H_{12}O_4$	961-29-5	C08650	256.1	255.1	119.1	-ive
10	Phloretin	Chalcones	$C_{15}H_{14}O_5$	60-82-2	C00774	274.1	273.1	167	-ive
11	Sieboldin	Chalcones	$C_{21}H_{24}O_{11}$	18777-73-6	-	452.1	451.13	289.07	-ive
12	Naringenin chalcone	Chalcones	$C_{15}H_{12}O_5$	25515-46-2	C06561	272.1	271.1	151	-ive
13	(-)-Catechin	Flavanols	$C_{15}H_{14}O_6$	18829-70-4	C14079	290.1	289.1	123	-ive
14	(-)-Gallocatechin gallate	Flavanols	$C_{22}H_{18}O_{11}$	4233-96-9	-	458.1	457.1	169	-ive
15	(-)-Catechin gallate	Flavanols	$C_{22}H_{18}O_{10}$	130405-40-2	-	442.1	441.1	169	-ive
16	(-)-Epigallocatechin	Flavanols	$C_{15}H_{14}O_7$	970-74-1	C12136	306.1	307.1	139	+ive
17	(-)-Epicatechin	Flavanols	$C_{15}H_{14}O_6$	490-46-0	C09727	290.1	289.1	109	-ive
18	(-)-Gallocatechin	Flavanols	$C_{15}H_{14}O_7$	3371-27-5	-	306.1	305.06	125.04	-ive
19	Bavachinin	Flavanones	$C_{21}H_{22}O_4$	19879-30-2	-	338.2	337.1	119.1	-ive
20	Bavachin	Flavanones	$C_{20}H_{20}O_4$	19879-32-4	-	324.1	323.1	119.1	-ive
21	Isosakuranin	Flavanones	$C_{22}H_{24}O_{10}$	491-69-0	-	448.1	447.1	285.1	-ive
22	Isosakuranetin	Flavanones	$C_{16}H_{14}O_5$	480-43-3	C05334	286.1	285.1	164	-ive
23	Liquiritigenin	Flavanones	$C_{15}H_{12}O_4$	578-86-9	C09762	256.1	255.1	119.1	-ive
24	Hesperidin	Flavanones	$C_{28}H_{34}O_{15}$	520-26-3	C09755	610.2	609.2	301.1	-ive
25	Hesperetin	Flavanones	$C_{16}H_{14}O_6$	520-33-2	C01709	302.1	301.1	164	-ive
26	Eriocitrin	Flavanones	$C_{27}H_{32}O_{15}$	13463-28-0	C09732	596.2	595.2	287.1	-ive
27	Eriodictyol	Flavanones	$C_{15}H_{12}O_6$	552-58-9	C05631	288.1	287.1	135	-ive
28	Narirutin	Flavanones	$C_{27}H_{32}O_{14}$	14259-46-2	C09793	580.2	579.2	271.1	-ive
29	Silibinin	Flavanonols	$C_{25}H_{22}O_{10}$	22888-70-6	C07610	482.1	481.1	301	-ive
30	Taxifolin 7-O-rhamnoside	Flavanonols	$C_{21}H_{22}O_{11}$	137592-12-2	-	450.1	449.1	285	-ive
31	Isoorientin	Flavone glycosides	$C_{21}H_{20}O_{11}$	4261-42-1	C01821	448.1	447.1	327.1	-ive
32	Vitexin	Flavone glycosides	$C_{21}H_{20}O_{10}$	3681-93-4	C01460	432.1	431.1	311.1	-ive
33	3'-Methoxypuerarin	Flavone glycosides	$C_{22}H_{22}O_{10}$	117047-07-1	-	446.1	445.2	325.1	-ive
34	Orientin	Flavone glycosides	$C_{21}H_{20}O_{11}$	28608-75-5	C10114	448.1	447.1	327.1	-ive
35	Chrysosplenetin	Flavones	$C_{19}H_{18}O_8$	603-56-5	C10030	374.1	373.1	343	-ive
36	Luteolin	Flavones	$C_{15}H_{10}O_6$	491-70-3	C01514	286.0	285	133	-ive
37	Linarin	Flavones	$C_{28}H_{32}O_{14}$	480-36-4	-	592.2	591.17	283.06	-ive
38	Sakuranetin	Flavones	$C_{16}H_{14}O_5$	2957-21-3	C09833	286.1	285.1	165	-ive
39	Diosmetin	Flavones	$C_{16}H_{12}O_6$	520-34-3	C10038	300.1	299.1	284.1	-ive
40	Apigenin 7-glucoside	Flavones	$C_{21}H_{20}O_{10}$	578-74-5	-	432.1	431.1	268	-ive
41	Jaceosidin	Flavones	$C_{17}H_{14}O_7$	18085-97-7	-	330.1	329.1	299	-ive
42	Diosmin	Flavones	$C_{28}H_{32}O_{15}$	520-27-4	C10039	608.2	607.2	299.1	-ive
43	Apigenin	Flavones	$C_{15}H_{10}O_5$	520-36-5	C01477	270.1	269.1	117	-ive
44	Apigenin-7-glucuronide	Flavones	$C_{21}H_{18}O_{11}$	29741-09-1	-	446.1	445.1	269.1	-ive
45	Wogonin	Flavones	$C_{16}H_{12}O_5$	632-85-9	C10197	284.1	283.1	163	-ive
46	Eupatorin	Flavones	$C_{18}H_{16}O_7$	855-96-9	-	344.1	343.1	328.1	-ive
47	5-O-Demethylnobiletin	Flavones	$C_{20}H_{20}O_8$	2174-59-6	-	388.1	389.1	359.1	+ive
48	Homoplantaginin	Flavones	$C_{22}H_{22}O_{11}$	17680-84-1	C17762	462.1	461.1	283	-ive
49	Galangin	Flavones	$C_{15}H_{10}O_5$	548-83-4	C10044	270.1	269	211	-ive
50	Narcissin	Flavones	$C_{28}H_{32}O_{16}$	604-80-8	-	624.2	623.2	315.1	-ive
51	Cynaroside	Flavones	$C_{21}H_{20}O_{11}$	5373-11-5	-	448.1	447.1	285	-ive
52	Scutellarin	Flavones	$C_{21}H_{18}O_{12}$	27740-01-8	-	462.1	461.1	285.1	-ive
53	Nobiletin	Flavones	$C_{21}H_{22}O_8$	478-01-3	C10112	402.1	403.1	388.1	+ive
54	Baimaside	Flavonols	$C_{27}H_{30}O_{17}$	18609-17-1	C12667	626.1	625.1	300	-ive
55	Quercimeritrin	Flavonols	$C_{21}H_{20}O_{12}$	491-50-9	C12639	464.1	463.09	175.1	-ive
56	Astragalin	Flavonols	$C_{21}H_{20}O_{11}$	480-10-4	C12249	448.1	447.1	284	-ive
57	Rutin	Flavonols	$C_{27}H_{30}O_{16}$	153-18-4	C05625	610.2	609.1	300	-ive
58	Spiraeoside	Flavonols	$C_{21}H_{20}O_{12}$	20229-56-5	-	464.1	463.1	301	-ive
59	Tiliroside	Flavonols	$C_{30}H_{26}O_{13}$	20316-62-5	-	594.1	593.1	285	-ive
60	Quercitrin	Flavonols	$C_{21}H_{20}O_{11}$	522-12-3	C01750	448.1	447.1	300	-ive
61	3,7,4'-Trihydroxyflavone	Flavonols	$C_{15}H_{10}O_5$	2034-65-3	C10037	270.1	271.1	137	+ive
62	Avicularin	Flavonols	$C_{20}H_{18}O_{11}$	572-30-5	-	434.1	433.1	300	-ive
63	Isorhamnetin 3-O-glucoside	Flavonols	$C_{22}H_{22}O_{12}$	5041-82-7	-	478.1	477.1	314.05	-ive
64	Typhaneoside	Flavonols	$C_{34}H_{42}O_{20}$	104472-68-6	-	770.2	769.2	314	-ive
65	Kaempferol 3-neohesperidoside	Flavonols	$C_{27}H_{30}O_{15}$	32602-81-6	-	594.2	593.2	284	-ive
66	Flavonol	Flavonols	$C_{15}H_{10}O_3$	577-85-5	C01495	238.1	239.1	165.1	-ive
67	Miquelianin	Flavonols	$C_{21}H_{18}O_{13}$	22688-79-5	-	478.1	477.1	301	-ive
68	Icaritin	Flavonols	$C_{21}H_{20}O_6$	118525-40-9	-	368.1	367.1	297	-ive

**69**	Afzelin	Flavonols	$C_{21}H_{20}O_{10}$	482-39-3	C16911	432.1	431.1	285	-ive
**70**	Isorhamnetin	Flavonols	$C_{16}H_{12}O_{7}$	480-19-3	C10084	316.1	315.1	300	-ive
**71**	Kaempferitrin	Flavonols	$C_{27}H_{30}O_{14}$	482-38-2	C16981	578.2	577.1	431.1	-ive
**72**	Hyperoside	Flavonols	$C_{21}H_{20}O_{12}$	482-36-0	-	464.1	465.1	314.9	+ive
**73**	6"-O-Acetylglycitin	Isoflavones	$C_{24}H_{24}O_{11}$	134859-96-4	-	488.1	487.1	282.1	-ive
**74**	Glabridin	Isoflavones	$C_{20}H_{20}O_{4}$	59870-68-7	C10421	324.1	323.1	201.1	-ive
**75**	2'-Hydroxydaidzein	Isoflavones	$C_{15}H_{10}O_{5}$	7678-85-5	C02495	270.1	269	225.1	-ive
**76**	Tectorigenin	Isoflavones	$C_{16}H_{12}O_{6}$	548-77-6	C10534	300.1	299.1	240	-ive
**77**	Daidzin	Isoflavones	$C_{21}H_{20}O_{9}$	552-66-9	C10216	416.1	415.1	252.1	-ive
**78**	Prunetin	Isoflavones	$C_{16}H_{12}O_{5}$	552-59-0	C10521	284.1	283.1	268	-ive
**79**	Licoisoflavone A	Isoflavones	$C_{20}H_{18}O_{6}$	66056-19-7	C10486	354.1	353.1	284	-ive
**80**	Formononetin	Isoflavones	$C_{16}H_{12}O_{4}$	485-72-3	C00858	268.1	267.1	252.1	-ive
**81**	7-Methoxyisoflavone	Isoflavones	$C_{16}H_{12}O_{3}$	1621-56-3	C15616	252.1	253.1	210.1	+ive
**82**	Puerarin	Isoflavones	$C_{21}H_{20}O_{9}$	3681-99-0	C10524	416.1	415.1	295.1	-ive
**83**	Kurarinone	Other flavonoids	$C_{26}H_{30}O_{6}$	34981-26-5	C17446	438.2	437.2	161.1	-ive
**84**	Syringaldehyde	Phenonic acids	C9H10O4	134-96-3	-	182.1	181.1	151	-ive
**85**	Mangiferin	Xanthones	$C_{19}H_{18}O_{11}$	4773-96-0	C10077	422.1	421.1	331	-ive

Q1: parent ion; Q3: Daughter ion. CAS: Chemical Abstracts Service (a unique numeric identifier for chemical substances or molecular structures). KEGG (Kyoto Encyclopedia of Genes and Genomes) ID: A database, which is a unique identifier that begins with the letter "C" and is followed by five digits. Da: stands for Dalton.
